# Diabetes Mellitus and Its Metabolic Complications: The Role of Adipose Tissues

**DOI:** 10.3390/ijms22147644

**Published:** 2021-07-16

**Authors:** Lowell Dilworth, Aldeam Facey, Felix Omoruyi

**Affiliations:** 1 Department of Pathology, Mona Campus, University of the West Indies, Kingston 7, Jamaica; lowell.dilworth02@uwimona.edu.jm; 2 Mona Academy of Sport, Mona Campus, University of the West Indies, Kingston 7, Jamaica; aldeam.facey02@uwimona.edu.jm; 3 Department of Life Sciences, Texas A&M University, Corpus Christi, TX 78412, USA

**Keywords:** diabetes mellitus, adipose, insulin resistance

## Abstract

Many approaches have been used in the effective management of type 2 diabetes mellitus. A recent paradigm shift has focused on the role of adipose tissues in the development and treatment of the disease. Brown adipose tissues (BAT) and white adipose tissues (WAT) are the two main types of adipose tissues with beige subsets more recently identified. They play key roles in communication and insulin sensitivity. However, WAT has been shown to contribute significantly to endocrine function. WAT produces hormones and cytokines, collectively called adipocytokines, such as leptin and adiponectin. These adipocytokines have been proven to vary in conditions, such as metabolic dysfunction, type 2 diabetes, or inflammation. The regulation of fat storage, energy metabolism, satiety, and insulin release are all features of adipose tissues. As such, they are indicators that may provide insights on the development of metabolic dysfunction or type 2 diabetes and can be considered routes for therapeutic considerations. The essential roles of adipocytokines vis-a-vis satiety, appetite, regulation of fat storage and energy, glucose tolerance, and insulin release, solidifies adipose tissue role in the development and pathogenesis of diabetes mellitus and the complications associated with the disease.

## 1. Introduction

Diabetes mellitus is a metabolic disease that is characterized by hyperglycemia associated with alterations in carbohydrate, protein, and fat metabolism. There are three major types of the disease: Type 1 diabetes, type 2 diabetes, and gestational diabetes. In type 1 diabetes mellitus, absolute insulin deficiency is associated with autoimmune destruction of the pancreatic beta-cells. Type 2 diabetes mellitus is a heterogeneous disorder characterized by insulin resistance with varying degrees of insulin secretory defects, followed by reduced insulin secretion from the pancreas (pancreatic beta-cell dysfunction). Gestational diabetes is associated with pregnancy, due to temporary intolerance of glucose utilization. The abnormality in glucose metabolism usually returns to normal after delivery. The elevated production of antagonists to insulin during pregnancy results in increased secretion and release of insulin. A failing pancreas may be unable to meet this heightened demand for insulin, which results in gestational diabetes. Diabetic patients are unable to metabolize glucose efficiently and cannot synthesize fatty acids and triglycerides from carbohydrates or amino acids, due to the failure of insulin secretion or action. Since the cells cannot detect and absorb glucose in the blood, enzymes in the glycolytic, lipogenic, and pentose phosphate pathways are suppressed, while gluconeogenic, glycogenolytic, and lipolytic activities are elevated, reversing the metabolic pathway in nondiabetic individuals. The increased risk of morbidity and mortality from vascular complications in diabetic patients is linked to genetic factors, elevated glucose levels, hypertension, obesity, oxidative stress, blood lipid disorders, and smoking [1]. The chronic hyperglycemic condition alters cell membrane permeability to cations and transmembrane potential [2]. Due to constant oxidative stress in diabetic cells, hyperpolarization is responsible for the long-term complications of diabetes [3,4,5]. Sozmen et al. [6] reported that uncontrolled diabetes is responsible for glucose auto-oxidation, nonenzymatic protein glycation, and activation of the polyol pathway with increased oxidative stress. In type 2 diabetes, two primary pathophysiological mechanisms are involved: (a) Insulin resistance in skeletal muscle and liver, and (b) defective insulin secretion from the beta cells of the pancreas [7]. Accumulating evidence also suggests a strong link between the risk of type 2 diabetes and genetic factors [8]. There are over 463 million individuals currently living with diabetes mellitus, and it is expected to increase to 578 million by 2030 [9]. There is no cure for the disease, and treatment goals for diabetes mellitus include restoring the normal metabolic pathways and reversing dyslipidemia. Current treatment consists of oral glycemic agents, daily insulin injections, regulation of diet, or physical activity. This review will highlight the role of adipose tissue in the pathogenesis of diabetes and the complications associated with the disease.

### 1.1. Insulin Resistance

Insulin resistance is commonly associated with type 2 diabetes; however, recent studies reported insulin resistance in type 1 diabetes [10]. During insulin resistance, the secretion of the hormone from the pancreatic islet cells cannot trigger glucose uptake in metabolic tissues, leading to elevated blood glucose and insulin levels. Type 2 diabetes begins with a progressive decline of insulin action. In this state, the body cannot efficiently utilize insulin for insulin-mediated glucose transport into tissues, resulting in the desensitization of insulin receptors. To compensate for insulin resistance, the body produces additional insulin to facilitate glucose homeostasis. Insulin resistance is a pathological condition with impaired sensitivity to insulin in target tissues. The elevated blood insulin in insulin resistance drives dyslipidemia, high blood pressure, and glucose metabolism alteration [11]. The disruption of signals that allows for the balance of satiety and hunger during insulin resistance promotes excess calorie consumption. However, the ability to store excess fat calories in adipose tissue is affected by the upregulation of inflammation and the development of insulin resistance with the resultant increase in free fatty acids (FFA) in circulation and other organs from the adipose tissue. Obesity is also commonly associated with insulin resistance. The underlying mechanisms of insulin resistance are multifaceted, including inflammation, endoplasmic reticulum stress, oxidative stress, and mitochondrial dysfunction [12,13,14,15]. Type 2 diabetes is believed to be a natural immune and inflammatory disease [16]. Inflammatory factors include immune-inflammatory cells, acute-phase reaction proteins, interleukins, adiponectin, resistin, macrophage migration inhibitory factor, coagulation factors, lipid components, sialic acid, and amyloid [17,18,19]. Intracellular serine/threonine kinases are activated by inflammation factors and catalyze the inhibitory phosphorylation of key proteins of the insulin signaling pathway, leading to insulin resistance [20]. Macrophages produce cytokines, and chemokine promotes systemic inflammation that damages the islet cells of the pancreas and upregulates insulin resistance in the liver, fat, and skeletal muscle tissue [21]. Insulin resistance affects the metabolism of glucose and lipids that promote an increase in the levels of free fatty acids with associated stimulation of the body to produce large amounts of reactive oxygen species (ROS) and reactive nitrogen species (RNS) [22,23]. Free radicals generated by cellular oxidative stress are highly reactive molecular species with one or more unpaired electron(s). Increase levels of free radicals can damage cellular proteins, lipids, and nucleic acids.

**Figure 1:**
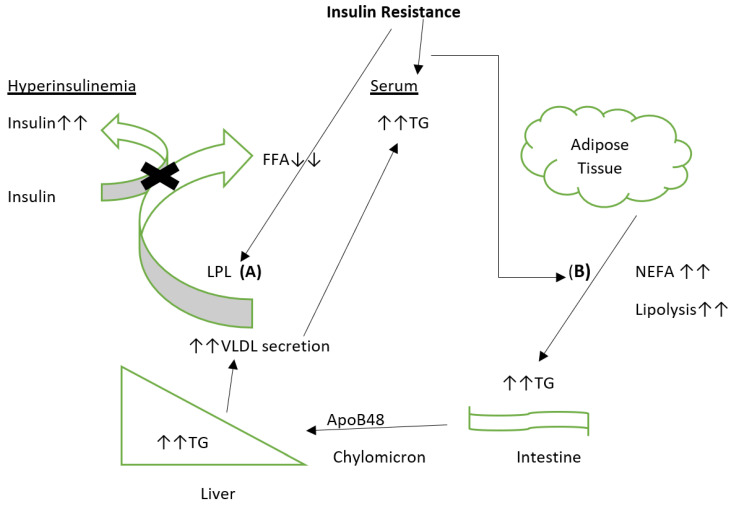
Insulin resistance leads to the diminished capacity of lipoprotein lipase (LPL) to metabolize very low-density-lipoprotein (VLDL) and other triglycerides (TG) rich particles to free fatty acids (FFA). It ultimately results in increased serum triglycerides because of blunted LPL activity (**A**). Chronic hyperinsulinemia facilitates VLDL production in states of insulin resistance. The suppressive effects of insulin are lost in insulin-resistant conditions, e.g., obesity or diabetes mellitus (DM). (**B**) Insulin resistance can lead to increased nonesterified fatty acid (NEFA) production via lipolysis, resulting in increased FFA flux to the liver, leading to increased VLDL production. Additionally, Fatty acids (FA) may increase, due to increased FA trapping by insulin-resistant adipocytes. Increased serum FA is associated with stimulation of the body to produce large amounts of reactive oxygen species.

On the other hand, antioxidants are substances that inhibit oxidative damage associated with free radicals. Matough et al. [24] demonstrated that type 2 diabetes induces free radicals’ generation while suppressing cellular antioxidant defenses. DNA damage, due to oxidative stress, promotes the activation of DNA repair enzyme, poly-ADP-ribose polymerase-1 (PARP-1), which inhibits glyceraldehyde 3-phosphate dehydrogenase (GAPDH) [25,26,27], and increases GAP levels, glucose, and other glycolytic intermediates like fructose-6-phosphate (F-6-P) and glucose-6-phosphate (G-6-P). The upregulation of these molecules in the cell activates different pro-oxidative pathways like advanced glycation end products (AGEs) and protein kinase C (PKC) pathways. Then the autooxidation of excess GAP leads to the production of hydrogen peroxide (H_2_O_2_), which further promotes oxidative stress [28]. Excessive intracellular and extracellular glucose induce spontaneous and nonenzymatic reactions with proteins. This nonenzymatic glycation of proteins involves complex reactions that produce advanced glycation end products (AGEs). Glucose auto-oxidation results in glyoxal formation, a precursor for advanced glycation end products that enhance cellular oxidative stress [29,30]. Oxidative stress also activates the nuclear factor-B (NF-B) signaling pathway by disrupting the mitochondrial structure, inducing apoptosis, and activating cellular inflammatory responses. Ma et al. [31] reported that oxidative stress interferes with insulin signaling that involves the phosphorylation of insulin receptor and insulin receptor substrate, activation of phosphatidylinositol 3-kinase (PI3K), and glucose transporter 4 (GLUT4) that then causes insulin resistance. Overall, insulin resistance plays a prominent role in developing type 2 diabetes and related complications, and therapeutic improvement in the cellular antioxidant activity may be protective against ROS-mediated damage. The biochemical role of adipose tissue in insulin resistance and further implications for diabetes mellitus cannot be understated and will be explored in further sections. The proposed mechanisms of insulin resistance are highlighted in Figure 1.

### 1.2. Adipose Tissue and the Development of Insulin Resistance

The two main types of adipose tissue are brown adipose tissue (BAT) and white adipose tissue (WAT), with beige subsets more recently identified. Functionally, they are essentially antagonistic. BAT is highly specialized, well-vascularized, and is located mainly around organs and tissues of the cardiovascular system, around solid organs, including kidneys, liver, adrenals, pancreas, between the shoulder blades, and in the armpits mediastinum and along the spine [32]. In the human context, BAT is used by newborns to generate heat through a process called nonshivering thermogenesis. For this to occur in BAT, there is uncoupling from normal mitochondrial metabolism generating heat and serving as a protective mechanism against hypothermia during the initial hours following birth [33]. BAT constitutes about 10% of the full-term newborn body weight, but the reserves are nonrenewable [34]. The brown adipose tissue is believed to be a potential therapeutic target for obesity and related metabolic disorders [35].

On the other hand, WAT may be generally classified based on location, primarily defined as subcutaneous and visceral/omental located intra-abdominally. The location allows for these fat deposits to also provide mechanical support in fingers and toes, the heels’ fat pads, and the periorbital fat supporting the eyes [36]. WAT is the predominant adipose tissue in humans, and the visceral depots in humans are associated with diabetes and cardiovascular diseases [37]. However, WAT is known to encompass the majority of body fat, its major role in the storage of surplus dietary triglycerides may expand via cellular hypertrophy with developing obesity [38]. Some studies indicate that healthy adipocytes comprise healthy WAT. The white adipose tissue tends to be small and has a high tendency towards mitochondrial oxidative phosphorylation and *de novo* lipogenesis, and triglycerides/fatty acid cycling [39]. Based on the lipotropic benefits of good WAT, patients with high percentages of this tissue should theoretically be less susceptible to dyslipidemias. Of note, WAT can be considered ‘unhealthy’ if there is excess or insufficient storage of lipid droplets that may lead to dyslipidemia, insulin resistance, and diabetes mellitus [40]. Overall, white adipose tissue seems to play critical roles in adipose endocrine function, communication, and insulin sensitivity.

The most recently identified type of adipose tissue is referred to as beige adipose tissue. This tissue results from the deposition of BAT within WAT deposits and may originate from white to brown differentiation or from de novo differentiation from specific precursor cells [41,42]. There are some similarities observed between WAT and beige adipose tissue, e.g., hypotriglyceridemic properties. However, both tissues show distinction, especially regarding the expression of genes involved in transcription, metabolism, and inflammation [41]. Upregulation of beige adipose tissue production is thought to occur, due to adrenergic signals and cold exposure. Other factors contributing to this induction include diet, genetics, and the anatomical location of the adipose tissue [43]. De novo differentiation can occur once progenitor cells within WAT are induced to differentiate into beige adipocytes [42]. Since beige adipocytes are inducible, there seems to be a constant relative flux between inactive and active beige cells, with cells reverting to a unilocular dormant phenotype upon warming [44]. Recent research has focused on brown and beige adipocytes considering their ability to counteract various metabolic and inflammatory diseases, including obesity and type 2 diabetes, via modulating specific molecular mechanisms.

Our current understanding of adipocytes and adipose tissue has recently expanded, mainly related to obesity-related complications. In addition to our knowledge of adipocytes releasing hormones, the fatty tissue also releases effectors, such as exosomes, microRNAs (miRNA), lipids, and inflammatory cytokines that affect systemic metabolic responses. Fatty tissue in organ and systemic levels serves as an energy reservoir, controls lipid mobilization [45], and plays a significant role in energy regulation and glucose homeostasis [46], comprising pre-adipocytes, macrophages, and endothelial cells, fibroblasts, and leucocytes. During energy surplus, body fat is stored as neutral triglycerides and released as fatty acids for energy needs [47]. Adipose tissue can also serve as an endocrine organ by secreting adipokines like leptin, adiponectin, visfatin, apelin, vaspin, hepcidin, chemerin, and omentin that are metabolically active in muscle, liver, pancreas, and brain [48,49,50].

### 1.3. Immune and Endocrine Functions of Adipose Tissue

Adipose tissue also has an immune system that is important for tissue homeostasis. The pathophysiological features of type 2 diabetes include insulin resistance, glucose intolerance, hyper-insulinemia, hyperglycemia, and diabetic dyslipidemia. Impaired glucose handling by adipocytes, hepatocytes, and muscle cells is linked to inflammatory pathways that interfere with insulin production and insulin signaling [51]. Interleukin-4 and interleukin-10 are critical regulatory cytokines to keep inflammation at bay as adipose tissue remodeling occurs during nutrient intake and energy expenditure. Among the pro-inflammatory cytokines, tumor necrosis factor-alpha is one of the most important pro-inflammatory mediators involved in developing insulin resistance. It is mainly produced in adipocytes and peripheral tissues and induces tissue-specific inflammation and activation of various transcriptional mediated pathways [18]. The cluster differentiation 40 (CD40) is a member of the tumor necrosis factor (TNF) receptors’ superfamily. The CD40–CD40L (CD40 ligand) pathway is essential in mediating various immune and inflammatory responses. In addition to the membrane-bound form (mCD40L), the soluble CD40L also exists (sCD40L) that is derived from activated platelets and T cells [52]. The interactions of CD40–CD40L are needed in many immune processes that involve the production of chemokine and cytokine, B cell activation, co-stimulation, immunoglobulin isotype switching, and memory cell formation [52,53]. Missiou et al. [54] reported that CD40L signaling in adipose tissue increases the expression of pro-inflammatory mediators. Elevated blood glucose and insulin upregulate the expression of membrane-bound CD40L on circulating platelets. The subsequent boost to the production of platelet-leukocyte aggregates and leukocyte-endothelium interactions enhances vascular inflammation [55,56]. Poggi et al. [57] showed that stimulation with recombinant CD40L impaired insulin-induced glucose uptake in adipocytes by down-regulating the expression of insulin receptor-1 (IRS-1) and glucose transporter-4 (GLUT-4). They proposed that CD40L may be a direct regulator of insulin and glucose tolerance. Therapeutically, targeted inhibition of CD40L-CD40 signaling may be a promising target for improving adipose tissue and vascular inflammation, which may reduce vascular complications associated with type 2 diabetes [58].

## 2. Adipose Tissue as an Endocrine Organ

Adipocytes serve a role in immunity, metabolism, cardiovascular function, and reproduction. These lipid-rich cells secrete a variety of proteins that serves as regulatory hormones [59]. White adipose tissue demonstrates its energy regulation and an endocrine function that mediates pathological and physiological actions [60]. The significance of white adipose tissues to endocrine function is demonstrated by its dysregulation, which causes obesity or lipoatrophy. Effective treatment of obesity is a global interest that has led researchers to study adipocyte-derived hormones and inflammatory cytokines called adipocytokines [61]. These include leptin, adiponectin, interleukin 6, resistin, tumor necrosis factor-alpha, acylation stimulating protein, among many others [62,63,64,65]. These hormones and cytokines have immune, cardiovascular, metabolic, and endocrine functions [66]. The energy status of adipocytes determines these adipocytokines’ secretion, which regulates appetite, glucose, and lipid metabolism [67]. Leptin, adiponectin, and resistin are the most frequently referenced adipocytokines.

## 3. Leptin

**Figure 2:**
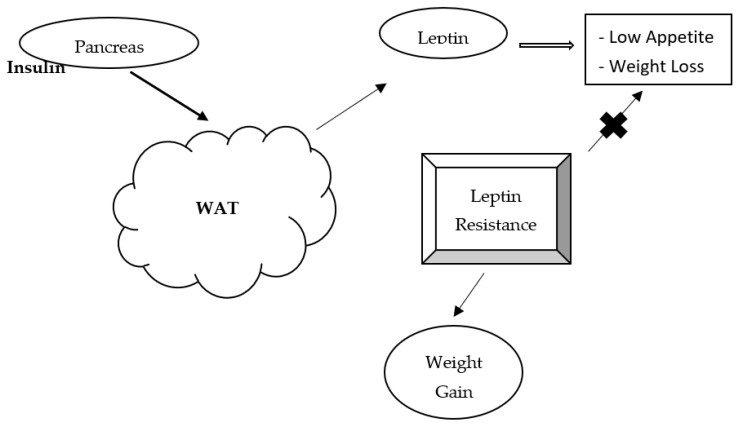
Insulin stimulates leptin synthesis and secretion from white adipose tissue. Increased serum leptin results in reduced appetite, food intake, and eventual weight loss. High serum leptin negatively impacts insulin secretion. In leptin-resistant patients, there is no suppression of appetite—eventually resulting in weight gain over time.

Zhang et al. [68] discovered the leptin molecule, and since its discovery, several studies have been published investigating its role in human metabolism. It is a 16-kDa polypeptide hormone mainly synthesized and secreted by white adipocytes and is integral in food intake regulation by decreasing appetite, modulating energy expenditure, and increasing sympathetic nervous activity [69,70]. These actions lead to appetite suppression, and hence, its consideration as an anti-obesity hormone. Leptin regulates neuroendocrine function, inflammation, endothelial dysfunction, and nutrient utilization [59,71,72]. It also plays a key role in regulating body weight and subsequently fat deposition by exerting its effects on hypothalamic receptors, resulting in appetite suppression [73]. Since leptin levels decrease during fasting, it is expected that increased leptin concentrations should correlate with decreased weight and vice versa. However, in some instances, this is not the case as serum leptin levels can correlate with body fat, a scenario thought to be caused by leptin resistance [74,75]. In this scenario, the ability of leptin to reduce appetite is somehow nullified. See Figure 2 for a diagram outlining the mechanism of leptin resistance. While studies assessing the correlation between leptin and diabetes mellitus are sparse, a predictive role of circulating serum leptin on reducing insulin sensitivity over time was observed in nondiabetic men [76]. Other studies demonstrate that insulin resistance and elevated plasma leptin levels are associated regardless of body weight [77]. Interestingly, animal studies show that leptin reduces hepatic glucose production, increases tissue glucose uptake, modulates IGF binding protein 2 (IGFBP2), and reduces blood glucose in mice [78,79]. More studies are needed in the area, especially in clarifying the role of leptin on insulin sensitivity in diabetic patients.

## 4. Adiponectin

Adiponectin is one of the most abundant adipocyte-secreting adipokines and has shown a negative correlation with obesity [80]. It exerts antidiabetic effects and modulates insulin resistance by stimulating lipid oxidation and anti-inflammatory responses [81]. Adiponectin seems to play similar roles in mammalian metabolism to leptin, with numerous studies on these hormones highlighting their roles in adiposity. A difference between both hormones is that in the acute setting, fasting reduces circulating leptin, while serum adiponectin concentration tends to increase with fasting [82,83]. While research associates adipokines, such as resistin and retinol-binding protein 4 with decreased insulin sensitivity, leptin and adiponectin are shown to have the opposite effect [84]. Other studies highlighting the positive effects of adiponectin on insulin sensitivity and secretion have led to the hormone being referred to as the antidiabetic adipokine [85,86]. A recent study shows that high adiponectin levels are associated with decreased type 2 diabetes (T2D) risk in the Chinese population. The hormone may be considered for use as a predictive marker of Type 2 DM [87]. Other recent research also noted a significantly lower serum concentration of adiponectin in patients living with type 2 diabetes mellitus [88]. Adiponectin was also lower in patients with higher body mass index values when compared with other patients where other parameters are considered [89]. Adiponectin, therefore, may predict the likelihood of developing type 2 diabetes mellitus and could be a therapeutic parameter. These molecules are considered metabolically beneficial based on their mechanisms of action, and decreased levels are observed in obesity associated with insulin resistance [90]. Metabolic dysfunction, due to insulin resistance in skeletal muscle, is thought to be a significant contributor to metabolic syndrome and diabetes mellitus [91]. Adiponectin activates autophagy in skeletal muscle cells, which in turn attenuates endoplasmic reticulum stress-induced reductions in insulin sensitivity in various tissues [92]. Adiponectin is thought to elicit these effects by upregulating a number of downstream signaling events. Studies have shown that adaptor protein, phosphotyrosine interacting with PH domain and leucine zipper 1 (APPL1), an adaptor protein, and insulin sensitizer, positively mediates adiponectin signaling in mammals by binding to them [93]. APPL1 is effective in the insulin-signaling pathway and is considered an important mediator of adiponectin-dependent insulin sensitization in skeletal muscle via a crosstalk mechanism between adiponectin and insulin [94]. While research has shown positive effects of adiponectin in modulating diabetes mellitus, further studies on the effects of adiponectin therapy in diabetics, especially regarding the biochemical roles of signaling molecules, are needed.

## 5. Resistin

Resistin is a polypeptide discovered in 2001, which measures approximately 12-kDa. Resistin counters insulin action and is proposed to link insulin resistance and obesity [63]. Researchers found that resistin mRNA expression in abdominal adipose tissues was 418% higher when compared to tissues in the thigh. It creates a link between the high risk of type 2 diabetes in persons with abdominal/central obesity [95]. It validates the use of the waist-hip ratio as an objective measure of the risk of type 2 diabetes and other non-communicable diseases. A summary of some adipocytokines and their functions is highlighted in Table 1.

## 6. Pathogenesis of Diabetic Complications

The underlying mechanisms in diabetic complications include genetic and epigenetic modifications, nutritional factors, and sedentary lifestyle [101,102]. The major microvascular complications in diabetes include nephropathy, neuropathy, and retinopathy. They are linked to chronic hyperglycemia, leading to the production of advanced glycation end products, generation of pro-inflammatory markers, and the induction of oxidative stress [103,104]. In healthy nondiabetic individuals, after a meal, there is usually an increase in blood glucose, resulting in insulin stimulation and release from the pancreas and an associated increase in insulin and glucose levels. The inhibition of lipid breakdown due to increased insulin promotes decreased blood fatty acid concentration. In type 2 diabetes, insulin’s inability to inhibit lipolysis and reduce blood levels of free fatty acids contributes to insulin resistance in muscles and the liver. Type 2 diabetics and obese nondiabetic individuals also have elevated triglyceride levels in muscles [105,106] and liver [107], which correlates with insulin resistance in these tissues. The oxidation of excess free fatty acids in type 2 diabetes results in (1) intracellular accumulation of acetyl CoA—a pyruvate dehydrogenase inhibitor, (2) elevated NADH/NAD ratio—a down regulator of the citric acid cycle, and (3) citrate accumulation—a potent inhibitor of phosphofructokinase. The inhibition of phosphofructokinase results in the build-up of glucose-6-phosphate that then inhibits hexokinase-2. The downregulation of glucose phosphorylation is accompanied by increased intracellular free glucose that prevents glucose transport in the cell through the GLUT4 transporter. Overall, the upregulation of blood-free fatty acid levels plus elevated triglyceride/fatty acyl COA levels in muscles, the liver, and beta cells of the pancreas result in muscle/hepatic insulin resistance development and the impairment of insulin secretion. There is the coexistence of elevated blood glucose and lipid levels in patients with poorly controlled diabetes. Sustained high blood glucose and lipids raise the risk for vascular complications [1]. The mechanisms include the formation and accumulation of advanced glycation end products, increased oxidative stress, protein kinase C activation, upregulation of hexosamine pathway, vascular inflammation, altered expression and action of hormone, growth factors, and cytokines [1]. Moreover, the chemical modification of lipoprotein in diabetes added to peroxidation and glycation is associated with the pathological mechanism that links lipid disorder with the complications of the disease [108,109].

## 7. Dyslipidemia in Type 2 Diabetes Mellitus

While all the mechanisms involved in developing diabetes mellitus are unknown, it is well established that obesity is an important factor that enhances the probability of type 2 diabetes mellitus (DM) development. In fact, it is theorized that obesity may account for between 80–85% of the risk of developing type 2 diabetes, an argument solidified by studies highlighting an 80-fold increase in the risk of developing DM by obese persons compared to those with normal body mass index (BMI) [110,111]. Obesity rates are increasing worldwide, with the main contributory factors being the interplay between environmental and genetic factors. The existence of individuals within an obesogenic environment leads to a positive energy balance [112]. The interplay between those factors and other genetic and endocrine factors, including the Ob gene and leptin, collectively play important roles in determining body weight [113]. Chronic caloric imbalance results in increased adipocyte hypertrophy and hyperplasia, leading to increased intracellular triglyceride storage. Excess adipogenesis eventually results in adipocytic abnormalities, dyslipidemia, and insulin resistance, due to increased production of angiotensin 2, resistin, TNF-α, interleukin 6, interleukin 1-β, and free fatty acids [114].

A wide array of lipid abnormalities is normally observed in obese patients. These include elevated triglyceride, VLDL, Apo B, and non-HDL-C levels along with low HDL-C [115,116,117]. It is important to note that while dyslipidemia should not be assessed in relation to adiposity alone, the associations between adiposity, dyslipidemia, and insulin resistance are well established. Insulin suppresses lipolysis, especially in the post-prandial state, by activating its downstream kinase Akt, which in turn results in the inhibition of the main driver of lipolysis, protein kinase A [118]. In chronic metabolic states that present as obesity and diabetes, tissues, including adipose tissue and skeletal muscle, become unresponsive to insulin. In these pathological states, there is usually an increase in insulin resistance, which blunts the inhibition of triglyceride lipolysis followed by an increase in triglyceride breakdown in adipose tissue, leading to increased fatty acid delivery to the liver [117,119]. In addition to that, the inhibition of Apo C-III expression by insulin is important in regulating the serum concentration of APO C-III-rich lipoproteins, including chylomicrons and VLDL. In states of insulin resistance and diabetes mellitus, Apo CIII levels will increase [120]. APO C-III inhibits cellular uptake of TG-rich lipids and is a known inhibitor of lipoprotein lipase (LPL), resulting in delayed clearance of TG-rich lipoproteins from circulation [121]. Additionally, APO C-III regulates triglyceride metabolism, negatively affects calcium handling and insulin sensitivity, and stimulates pancreatic beta-cell apoptosis, leading to insulin resistance and diabetes mellitus [122,123].

In patients with insulin resistance or diabetes mellitus, triglyceride-induced dyslipidemia appears to be a recurrent theme, with the triglyceride source being endogenous, exogenous, or both. Insulin resistance leading to compensatory hyperinsulinemia appears to be the main driver behind increased endogenous VLDL-TG production [124]. It may be due to indirect metabolic effects (including increased FFA concentration for increased TG production), or it could be due to the direct effects on insulin by the liver [125]. An exogenous increase in VLDL arises from disorders of chylomicron production, which is regulated by insulin and circulating FFA [126]. The central core of this mechanism lies in the actions of LPL. LPL, tethered to the luminal endothelial surface of vascular endothelium by proteoglycans, are integral in liberating fatty acids from TG-rich lipoproteins, including VLDL or remnants [127]. Since LPL is upregulated by insulin, any state of insulin resistance or diabetes mellitus will result in diminished LPL activity, elevated TG lipoproteins, and reduced HDL [128]. The impaired effect of insulin on LPL postprandially is thought to be an important contributor to atherogenic dyslipidemia described in insulin resistance syndrome and type 2 diabetes mellitus [129]. Conversely, low serum TG concentrations and high serum HDL are observed in patients with increased LPL activity with concurrent lowered risk of cardiovascular diseases (CVDs) in these patients [130].

## 8. Adipose Tissue and Metabolic Dysfunction

**Figure 3:**
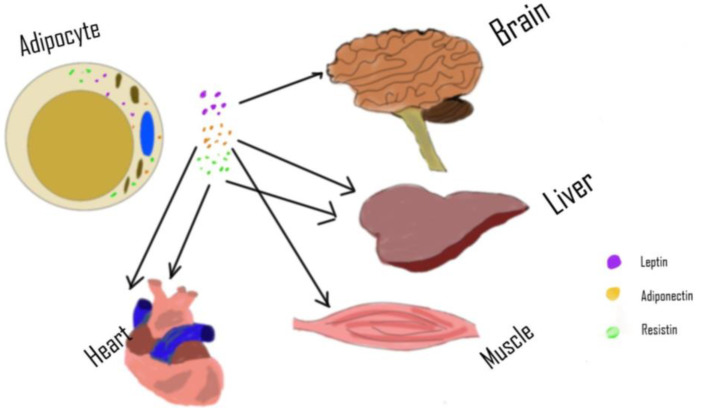
Lipid-filled adipocytes release the hormones adiponectin, leptin, and resistin, which target various tissues to affect their metabolic actions. Adiponectin targets the liver, skeletal muscles, bone, cartilage, heart, and adipose tissues. Leptin primarily targets the brain and central nervous system, while resistin targets adipose tissues, liver endothelium, and heart.

Metabolic dysfunction is the co-occurrence of parameters that negatively affect the cardiovascular system. These risk factors are interrelated and may share similar mechanisms. These parameters include insulin resistance, hypertension, dyslipidemia, and obesity [131]. All the parameters listed are associated with a large accumulation of adipose tissues. Therefore, adipose tissues’ adipocytokines are an important element when considering the progression of metabolic dysfunction and how to reverse the condition. Figure 3 highlights the main adipocytokines and the main organs from which they are derived.

Insulin resistance and an unfavorable lipid profile are usually associated with type 2 diabetes. These disorders are usually seen in conjunction with adipose tissue dysfunction [132]. In obesity, activated adipose tissue autophagy contributes to endocrine dysfunction [133]. Therefore, the presence of a large quantity of adipose tissue alone is not the cause of metabolic distress but also the product of these tissues. Adipocytes are actively involved in immune response, homeostasis, metabolizing steroids, and several other metabolic processes [134]. Increased numbers of adipocytes are associated with inflammation and other metabolic alterations that influence metabolic dysfunction development. There is a direct link between metabolic dysfunction and adipose tissue. It is believed that adipose tissue dysfunction is characterized by decreased insulin sensitivity, tissue inflammation, hypoxia, and increased apoptosis [135]. Dysfunction of adipose tissue, which may cause metabolic dysfunction, is caused by genetic, behavioral, and environmental factors [135]. The mechanism of action that links adipocytes with metabolic syndrome has been hypothesized to be the inability of adipose tissue to store excess lipids. There may be an abundance of adipocytes that are not able to expand and accommodate lipid molecules. This has been found to be closely linked to metabolic homeostasis [47].

## 9. Lipokines and Adipose Tissue

Adipose tissue has a significant role in the metabolic adaptations linked to disease development and prevention. Environmental factors like exercise and exposure to cold may also contribute to varying metabolic adaptation in white and brown adipose tissue [136,137,138,139,140,141]. Brown adipose tissue has greater metabolic activity than white adipose tissue; hence, the upregulation of brown adipose tissue accrues glucose tolerance and insulin sensitivity benefits [142,143,144]. Lipokines have emerged as a class of endocrine factors that are derived from adipose tissue. Some investigated lipokines, including palmitoleate, prostaglandins, lysophosphatidic acid (LPA), palmitoleate, 12,13-diHOME, fatty acid–hydroxy–fatty acids (FAHFAs), oxylipins, and N-acyl amino acids, are taking on increased significance due to their roles in carbohydrate metabolism, insulin resistance and lipid metabolism [136]. They are among the signal molecules from adipose tissue with involvement in the mediation of communication between adipose tissue and other metabolic tissues that may ultimately be an effective therapeutic target for metabolic disease. Lipokines are also products of other metabolic tissues [145,146,147]. Saturated fatty acids promote insulin resistance versus polyunsaturated fatty acids that do not affect insulin resistance but promote insulin sensitivity [148,149]. Palmitoleate is a lipokine because it is released from adipose tissue and affects distant organs [150]. The synthesis and secretion of the lipokine, palmitoleate by the adipose tissue results in the downregulation of liver lipid synthesis and the upregulation of skeletal muscle insulin action in vitro, demonstrating the potential role of palmitoleate mediation of the inter-tissue metabolism [136]. Hence increase in dietary intake of palmitoleate or the de novo synthesis may improve metabolic homeostasis. Exogenous palmitoleate has been linked to lipolytic stimulation, fatty acid esterification, mitochondrial beta-oxidation, upregulation of oxygen consumption, and GLUT-4 translocation. Although palmitoleate has been shown to promote insulin sensitivity [151,152], it is however not linked to a decrease in obesity [153]. Many lipokines are formed by adding fatty acid chains to other molecules like amino acids or even fatty acids themselves. Overall, there is a growing recognition of adipose-secreted endocrine lipids, lipokines that constitutes an important class of chemical messengers that may translate into therapeutic targets for the effective treatment of obesity, insulin resistance, type 2 diabetes, and other metabolic diseases [154]. We propose that lipokines play significant roles in the pathogenesis of diabetes mellitus and other metabolic disorders, due to the important roles they play in carbohydrate and lipid metabolism. More studies are needed in this area geared at identifying additional lipokines and unraveling their mechanism of action.

## 10. Adipose Tissue, Cholesterol Metabolism and Ethnicity

Adipose tissue in animals and humans contains a free and unesterified form of cholesterol [155,156,157]. About 93% or more of all cholesterol is stored in the unesterified form. Cholesterol enters the adipocyte by at least four distinct channels [158]:

- Via the LDL receptor,
- Through receptor-independent uptake of cholesterol,
- Via the VLDL receptor,
- Through the hydrolysis of triglyceride-rich lipoproteins.

Insulin presence results in the expansion of cellular triglyceride stores and reduced binding of lipoproteins, while the availability of catecholamines depresses cellular triglycerides with enhanced lipoprotein binding [158]. People with obesity-associated insulin resistance have changes in some key enzymes involved in HDL metabolism, like cholesteryl ester transfer protein, lecithin/cholesterol acyltransferase, hepatic lipase, and phospholipid transfer protein. However, partial cholesteryl ester transfer protein deficiency contributes to the ectopic accumulation of cholesteryl ester and triglyceride at their site of synthesis instead of being transported to storage droplets, which then disturbs lipid metabolic pathways because of their inaccessibility to hydrolytic enzymes [159]. Overall, the deficiency of cholesteryl ester transfer protein leads to abnormal triglycerides and cholesterol storage and downregulates the membrane ratio of free cholesterol/protein associated with the induction of insulin resistance and the changes in adipocytokines synthesis.

In obesity, adipocytes are overloaded, and they become dysfunctional leading to a decrease in the ability to store fats. Demographically, South Asians have been shown to have a significant increase in subcutaneous adipocyte size that has been linked with insulin resistance in nondiabetic individuals. The observed upregulation of fasting plasma levels of free fatty acids in South Asians compared to white Caucasians indicates adipocyte dysfunction in that population. However, the elevated plasma free fatty acid concentration was not suppressed even during the hyperinsulinemia-induced oral glucose tolerance test [160]. The inability of insulin to sufficiently inhibit lipolysis results in the excess efflux of free fatty acids, which may contribute to type 2 diabetes development in this population. Plasma leptins levels were also elevated in South Asians compared to Caucasian individuals and vice versa with adiponectin [161]. Overall, dysfunctional adipose tissue and inflammation may contribute to the South Asian phenotype of upregulated insulin resistance and type 2 diabetes development [162].

Similarly, a higher prevalence of obesity has been reported in the African population than European ancestry [163]. African ancestry has also been reported to have high insulin levels in the blood, more insulin resistance, and a greater risk for type 2 diabetes than European ancestry [163,164,165]. Eastwood et al. [166] reported that visceral adipose tissue may have more influence on the development of diabetes in South Asians than European men and concluded that overall truncal adiposity. However, in African Caribbeans, the levels of truncal subcutaneous fat were higher with potential toward the development of peripheral insulin resistance and the risk of diabetes even in the absence of high levels of visceral adipose tissue [166]. African ancestry has higher adipose tissue hypoxia than European ancestry, which may increase the levels of oxidative stress with an associated increase in circulating free fatty acids concentrations and reduced insulin sensitivity among Africans. Hence ethnic differences in insulin sensitivity and type 2 diabetes risk may be linked to the variation in the upregulated oxidative stress [163]. Of note, however, reactive oxygen species do not only cause oxidative stress. Still, they may also serve as signaling molecules that may promote health by either preventing or delaying chronic disease development that may accrue some health benefits [167]. The elevated circulating free fatty acids may not solely be derived from an impaired adipose tissue function, but could also be attributable to increased dietary fat intake [168]. However, excessive generation of reactive species can deplete the antioxidant defense system. Hence chronic exposure to high reactive oxygen species may lead to persistent inflammation and insulin resistance, due to decreased insulin signaling and impaired glucose and lipid metabolism in tissues [169,170]. In African women, observed higher circulating free fatty acids correlated with downregulation of insulin sensitivity [171]. Overall, a higher oxidative state in the adipose tissue may promote metabolic dysfunction [167]. Hence a rigorous study of oxidative stress in adipose tissue may elucidate the root cause of the prevalence of metabolic diseases and the development of effective therapeutic management and prevention among the African population.

## 11. Conclusions

The rates of obesity are increasing worldwide, with a projection of 700 million persons expected to be affected by 2045. With this trend and the impending impact of this metabolic disorder on the prevalence of diabetes mellitus, there has been enhanced focus on obesity and the spinoff metabolic complications which have or will adversely affect the quality of life. Adipose tissue is now considered an important metabolic and endocrine organ that plays crucial roles in whole-body insulin sensitivity and energy homeostasis. The expression and secretion of adipokines are tightly regulated by physiological and pathophysiological mechanisms, the disruption of which can lead to metabolic disorders, including diabetes mellitus. Adipocyte dysfunction in South Asian populations may stem from increased adipocyte size, resulting in spillover metabolic effects that indirectly contribute to insulin resistance and type 2 diabetes. From an Afro-Caribbean perspective, the prevalence of truncal fat may be a significant factor in the development of DM, even in instances of low visceral obesity. The incidence of adipose tissue hypoxia in Africans being higher than in Europeans could account for the increased oxidative stress and free serum fatty acids that may play a role in insulin resistance and higher rates of type 2 diabetes in persons of African ancestry. This review also reiterates that adipokines play essential roles in satiety, appetite, regulation of fat storage and energy, glucose tolerance, and insulin release, solidifying the important role that adipose tissue plays in the development and pathogenesis of diabetes mellitus. Further studies are needed to assess the molecular mechanisms that drive adipokine action and the implications for the prevention and management of diabetes mellitus in the clinical setting.

## Figures and Tables

**Table 1:** Summary of the primary function of key adipocytokines produced by white adipose tissue.

Adipocytokine	Primary Function	Source
Leptin	Regulates food intake	Minokoshi et al., 2002 [96]
Adiponectin	Regulates insulin sensitivity	Achari and Jain 2017 [94]
Resistin	Antagonize insulin action Links obesity with diabetes	Steppan et al., 2001 [97]
Interleukin 6	Involved in immune response to inflammation	Tanaka et al., 2014 [98]
Tumor Necrosis Factor	Multifunctional cytokine used by the immune system for cell signaling	Wang and Lin, 2008 [99]
Acylation Stimulating Protein	A fat storage factor that stimulates the synthesis and storage of triglycerides in adipocytes	Hlavatý and Kunesová, 2006 [100]

## Data Availability

Not applicable.
